# Influence of horse demographics, country of training and race distance on the rating of Thoroughbreds

**DOI:** 10.5194/aab-66-299-2023

**Published:** 2023-10-25

**Authors:** Eva Sobotková, Tomáš Kopec, Vladimír Mikule, Dana Kuřitková

**Affiliations:** Department of Animal Science, Mendel University in Brno, Zemědělská 1, Brno, 613 00, Czech Republic

## Abstract

The aim of the research was to assess how age, sex, sire, country of foaling, country of training and race distance influenced the international racing and performance of Thoroughbreds. The research was based on performance ratings of 6216 horses assigned by the International Federation of Racing Authorities between 2004 and 2022. The most common sex was stallion (58.54 %) and more than half of the population consisted of 3- and 4-year-old horses (54.68 %). The majority of the horses had the USA as their country of foaling (25.92 %) and also as their country of training (24.87 %). The sire with the largest number of offspring in the International Federation of Horseracing Authorities (IFHA) databases was Galileo (IRE) (193 horses). Four of the 10 most frequently represented sires belonged to the Sadler's Wells (USA) paternal line. The analysis of the statistics in the database as a whole established a significant ( p<0.001 ) influence of all observed factors. Stallions achieved a significantly higher rating (117.85) compared to geldings (117.17) and mares (117.13). The horses originating in Ireland achieved a statistically higher rating (117.99) than horses from Argentina, Australia, Brazil, New Zealand, a group of other countries designated “Others” and South Africa. Statistically conclusive differences were found between horses trained in Ireland (118.80) and all other countries except Great Britain and France. Five of the 10 sires with the best offspring rating belong to the Mr. Prospector (USA) paternal line.

## 1 Introduction

The Thoroughbred horse is a highly valued domestic animal population under strong selection for athletic phenotypes (McGivney et al., 2020). According to Rooney et al. (2018), Thoroughbred horses are finely tuned athletes with a high aerobic capacity relative to skeletal muscle mass, attributable to centuries of genetic selection for speed and stamina. The performance ability, constitution, health, character and temperament of this breed is assessed using a special performance test: flat racing (Jockey Club, 2017). The global leading authority for the international sport of Thoroughbred racing is the International Federation of Horseracing Authorities (IFHA), which was founded in 1993 and brings together 62 countries of the world. The World Thoroughbred Racehorse Ratings are the official end of season assessments of the top Thoroughbred racehorses. The numerical values (ratings) are based on the performance of horses in international flat races and take into account the quality of the opposition and the achievements of each horse (IFHA, 2022). The athletic potential and performance of a racehorse depend on a favourable environment as well as the inheritance of the optimal combination of DNA variants at loci that significantly affect exercise (Bower et al., 2012). Genetic factors and ways of making use of them have recently been addressed by several authors (Oki et al., 2005; Williamson and Beilharz, 1998; Mota et al., 2011; McGivney et al., 2020; Bakhtiari and Kashan, 2009; Sobczyńska and Lukaszewicz, 2004; Burns et al., 2006). Although the non-genetic factors that influence the performance of racehorses are still less well known, factors examined by most studies include sex, age, breed class, track condition, handicap weight and distance (More, 1999; Jiskrova et al., 2004; Gramm and Marksteiner, 2010; Klecel et al., 2021; Sobczyńska, 2007). An understanding of the role of these and other factors may assist trainers and others to optimize the performance of the horses under their care (More, 1999). The aim of this study was to confirm the hypothesis that selected factors have an effect on the performance of Thoroughbreds and that statistically significant differences in horse performance will be found in terms of age, sex, horse sire, race distance, country of foaling and country of training.

## 2 Materials and methods

The research was based on the performance ratings published by the International Federation of Horseracing Authorities (IFHA). The LONGINES World's Best Racehorse databases are based on the performance of horses running worldwide in elite flat races held during the designated period, taking into account the quality of the opposition and the achievements of each horse. The IFHA performance rating is an internationally recognized system and every specific value accurately represents the performance quality of each horse within the global population. Thanks to the unified system, it is possible to statistically analyse this number based on the distribution of horses into specific groups according to the monitored factors. The ratings are compiled by racing officials and handicappers (IFHA, 2022). The research was based on performance ratings of 6216 horses published by the International Federation of Racing Authorities between 2004 and 2022. Data from 2020 and 2021 may have been affected by the worldwide Covid situation. The following data were extracted from the IFHA database: horse's name, age, sex, country of foaling (suffix), country of training (suffix), racing season and rating points for each distance. Databases of the world's best racehorses, rated at least 115 or above, were supplemented with information on horse pedigrees from Pedigree Online's Thoroughbred Database (Pedigreequery, 2021). The official suffix system of the *General Stud Book* and the International Federation of Horseracing Authorities is used for the suffixes of horses and country abbreviations.

The data were processed in Excel, a statistical analysis was performed in Statistica 14, and the R statistical package R 4.2.1 was used. To evaluate the influence of selected factors on the rating, a general linear model (GLM, an analogue of ANOVA with a lognormal distribution) was used in the R programme. The dependent variable rating showed a significant deviation from the normal frequency distribution; therefore, a logarithmic transformation of the original rating values was used. The statistical significance of individual effects was assessed using the F test (ANOVA Table Type I). The null hypothesis was rejected in all cases at the significance level α=0.05 .

Sex, age, country of foaling, country of training, race distance and sire were treated as the main effects. For the purposes of the analysis, we combined underrepresented countries of foaling and countries of training into a group designated “Others (OTH)”.

The following model equation was used:

$$y_{ijklmn}=\mu +{\text{sex}}_{j}+{\text{age}}_{k}+{\text{cf}}_{l}+{\text{ct}}_{m}+{\text{dist}}_{n}+e_{ijklmn},$$

where $y_{ijklmn}$ is the evaluated quantity, μ is the overall average (mean) of the set, sex ${}_{j}$ is the effect of the j th sex ( j=3 ; stallion, mare, gelding), age ${}_{k}$ is the effect of the k th age ( k=8 ; 3–10 years), cf ${}_{l}$ is the effect of the l th country of foaling group ( l=12 ), ct ${}_{m}$ is the effect of the m th country of training group ( m=14 ), dist ${}_{n}$ is the effect of the n th group based on race distance ( n=5 ), and $e_{ijklmn}$ is the random residual error.

In order to evaluate the sire effect as well, we performed a second analysis. To increase objectivity, we excluded poorly represented sires (with less than 20 offspring).

$$y_{ijklmno}=\mu +{\text{sex}}_{j}+{\text{age}}_{k}+{\text{sire}}_{l}+{\text{cf}}_{m}+{\text{ct}}_{n}+{\text{dist}}_{o}+e_{ijklmno},$$

where $y_{ijklmo}$ is the evaluated quantity, μ is the overall average (mean) of the set, sex ${}_{j}$ is the effect of the j th sex ( j=3 ; stallion, mare, gelding), age ${}_{k}$ is the effect of the k th age ( k=8 ; 3–10 years), sire ${}_{l\;}$ is the effect of the l th sire ( l=63 ), cf ${}_{m}$ is the effect of the l th country of foaling group ( m=12 ), ct ${}_{n}$ is the effect of the m th country of training group ( n=14 ), dist ${}_{o}$ is the effect of the n th group based on race distance ( o=5 ), and $e_{ijklmo}$ is the random residual error.

Subsequently, the method of multiple comparisons by Scheffé's test was used to define significant differences caused by the individual effects. Scheffé's test is a post hoc test used in ANOVA. After ANOVA had been run and a significant F statistic had been obtained, Scheffé's test was then performed to determine which pairs of means were significant.

A multifactorial analysis of variance with interactions between individual factors was also part of the analysis. Unfortunately, it was not possible to evaluate the analysis objectively due to the absence or insufficient representation of data in individual levels of interaction.

Animal Care and Use Committee approval was not required for this study because the data were obtained from an existing database publicly accessible on the IFHA website (IFHA, 2022).

## 3 Results

The mean rating of racehorses in the IFHA databases in the monitored period was 117.57 with a standard deviation of 3.0458; the minimum was 115 and the maximum was 141 (Table 1).

**Table 1:** Descriptive statistics of the dataset.

	N	Median	Mean	SD	SEM	Min	Max
Rating	6216	117.00	117.57	3.0458	0.0386	115.00	141.00

SD: standard deviation; SEM: standard error of the mean.

The statistical analysis established a significant ( p<0.001 ) influence of all observed factors (Table 2).

**Table 2:** Results of GLM for age, sex, country of foaling, country of training and distance factors.

	Df	Sum sq	Mean sq	F value	Pr ( >F )	
Sex	2	0.0504	0.0252194	42.1572	$<2.2\times 10^{-16}$	${}^{***}$
Age	5	0.0194	0.0038764	6.4798	$5.13\times 10^{-6}$	${}^{***}$
Country of foaling	11	0.0813	0.0073947	12.3612	$<2.2\times 10^{-16}$	${}^{***}$
Country of training	13	0.0859	0.0066085	11.0469	$<2.2\times 10^{-16}$	${}^{***}$
Distance	4	0.0552	0.0137905	23.0524	$<2.2\times 10^{-16}$	${}^{***}$

Df: degrees of freedom; sum sq: predicted residual error of sum of squares; mean sq: mean square error; F : statistics; Pr: significance. ${}^{***}$ : p<0.001 .

### 3.1 The effect of the sex

*Evaluation of population.* Stallions account for more than half of the most successful horses (58.54 %), geldings for a further 26.25 %, and mares for only 15.20 % of the top-performing horses on average.

*Statistical analysis.* According to our results, stallions not only dominate international horse racing in terms of their number but also because their rating (117.85) is statistically highly significantly better than that of geldings and mares. On the other hand, there is no significant difference between mares and geldings, and their mean rating differs only slightly (117.13 and 117.17).

### 3.2 The effect of age

*Evaluation of population.* More than half of the horses (54.68 %) are 3 or 4 years old, and only 9.01 % are horses aged 7 years or older. During the monitored period, there were no significant fluctuations in the representation of horses of different ages, and their proportions in the databases of the best horses remained stable. The average age of all the horses that have achieved a rating of 115 is 4.43.

**Figure 1:**
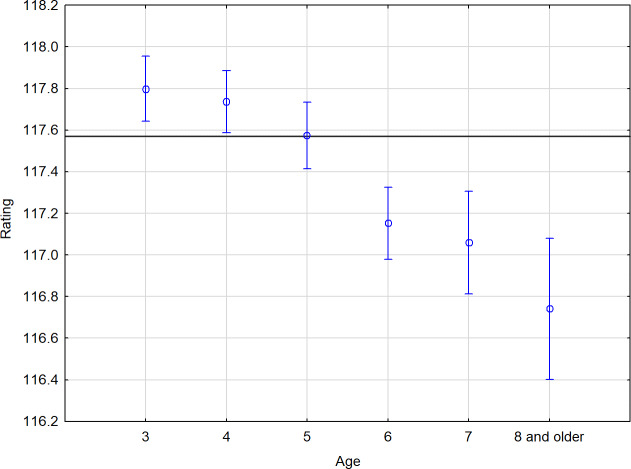
Performance summary of horses according to age. The horizontal line denotes the mean rating of the monitored population. The vertical bars represent the standard errors of differences (confidence interval 0.95).

*Statistical analysis.* Horses that were 3 to 4 years old achieved a statistically highly significantly better rating (117.80 and 117.74) than horses aged 6 years or older (117.08) (Fig. 1).

### 3.3 The effect of the country of foaling

*Evaluation of population.* The representation of individual countries of foaling has changed over the years. The most obvious trend is the gradual decrease in the number of horses from the USA from 37.75 % in 2004 to an average of around 21.00 % in recent years. There was also a decrease in the number of horses from Great Britain (from 17.65 % to 10.94 %) and Germany (from 3.43 % to 1.52 %). By contrast, an increase in the number of successful horses was achieved by Japan (from 7.84 % to 14.89 %), Australia (from 4.41 % to 13.98 %), New Zealand (from 0.98 % to 3.65 %) and South Africa (from 0.49 % to 3.34 %). No other trend of consistent development was observed, and other countries maintained their share of represented horses with minor fluctuations.

**Figure 2:**
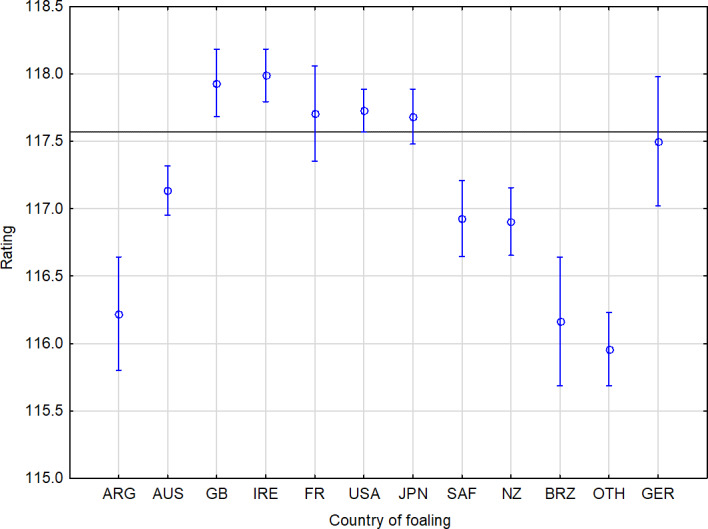
Performance summary of horses according to country of foaling. The horizontal line denotes the mean rating of the monitored population. The vertical bars represent the standard errors of differences (confidence interval 0.95).

*Statistical analysis.* Horses from the following countries achieved the best population average: Ireland (117.99), Great Britain (117.93), the USA (117.73), France (117.71) and Japan (117.68). Statistically, horses from Ireland, Great Britain and the USA have a significantly higher rating compared to those from Argentina (116.22), Australia (117.14), Brazil (116.16), New Zealand (116.90), the Others group (115.96) and South Africa (116.93). In addition, horses born in France and Japan have a statistically higher rating than horses from Argentina, Brazil and countries in the Others group (Table 3, Fig. 2).

**Table 3:** Results of Scheffé's test for country of foaling.

	Significant differences ( P<0.01 )	Significant differences ( P<0.05 )
IRE (117.99)	ARG, AUS, BRZ, NZ, OTH, SAF	
GB (117.93)	ARG, AUS, BRZ, NZ, OTH, SAF	
USA (117.73)	ARG, BRZ, OTH	AUS, NZ, SAF
FR (117.71)	OTH	ARG, BRZ
JPN (117.68)	OTH	ARG, BRZ

### 3.4 The effect of the country of training

*Evaluation of population.* In the observed period, there was an interesting development in the representation of horses by country of training. Among the most successful racehorses, the number of horses trained in the USA gradually decreased from 36.76 % in 2004 to 21.88 % in 2022. The representation of horses trained in France also decreased from 10.78 % to 6.08 %. The same trend applies to Germany (from 3.92 % to 1.21 %) and Great Britain (from 24.51 % to 19.76 %). On the other hand, there was a gradual increase in the number of horses from Japan (from 8.33 % to 15.81 %), Hong Kong (from 2.45 % to 5.17 %) and Australia (from 4.90 % to 16.41 %), and horses trained in South Africa, Argentina and New Zealand gradually appeared in the databases.

**Figure 3:**
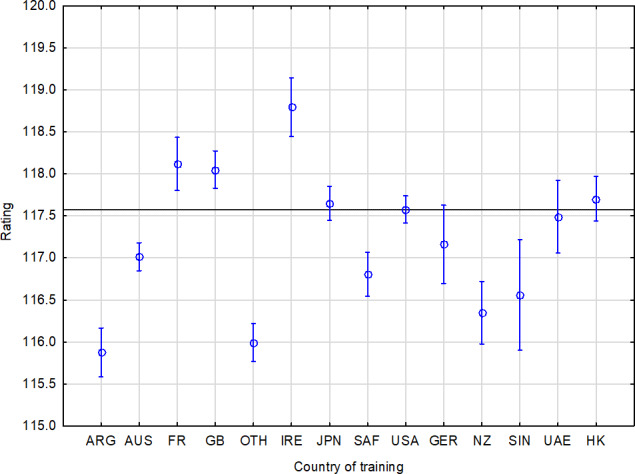
Performance summary of horses according to country of training. The horizontal line denotes the mean rating of the monitored population. The vertical bars represent the standard errors of differences (confidence interval 0.95).

*Statistical analysis.* Highly significant statistical differences were found between individual countries of training – see Table 4 and Fig. 3. The results of the best racehorses competing in recent years have demonstrated the superiority of Irish-trained horses. In connection with the proven success of Irish horses from the point of view of the country of foaling, this shows that Ireland is currently the most successful country in the racing industry in terms of the rating achieved.

**Table 4:** Results of Scheffé's test for country of training.

	Significant differences ( P<0.01 )
IRE (118.80)	ARG, AUS, GER, HK, JPN, NZ, OTH, SAF, SIN, UAE, USA
FR (118.12)	ARG, AUS, OTH, NZ, SAF, SIN
GB (118.05)	ARG, AUS, OTH, NZ, SAF, SIN
HK (117.70)	ARG, OTH
JPN (117.65)	ARG, OTH
USA (117.58)	ARG, OTH

### 3.5 The effect of the race distance

*Evaluation of population.* The most frequently represented race distance in the IFHA databases is the mile category (37.00 %), while a further 23.97 % of horses were successful at the intermediate distance and 20.95 % at the long distance. Sprint horses make up 13.69 % of the IFHA databases, and the least represented distance is the extended distance (4.39 %).

If we relate the age of horses to the individual distance categories, we find that the mile distance prevails for horses of all ages (always over 35.00 %), and the proportion of horses from all age categories that are successful at the intermediate distance is also stable (21 %–26.00 %). At the sprint distance, horses from older age categories are most frequently represented (61.82 % are horses aged 6 years and above), while 3-year-old horses are the least represented (8.9 %).

The analysis of the representation of individual genders showed that 47.09 % of the mares received a high rating at the mile distance, and at the extended distance only 3.30 % of top horses were mares. The only distance where more geldings (48.41 %) than stallions (41.13 %) started is the sprint; at all other distances, the stallions dominate.

**Figure 4:**
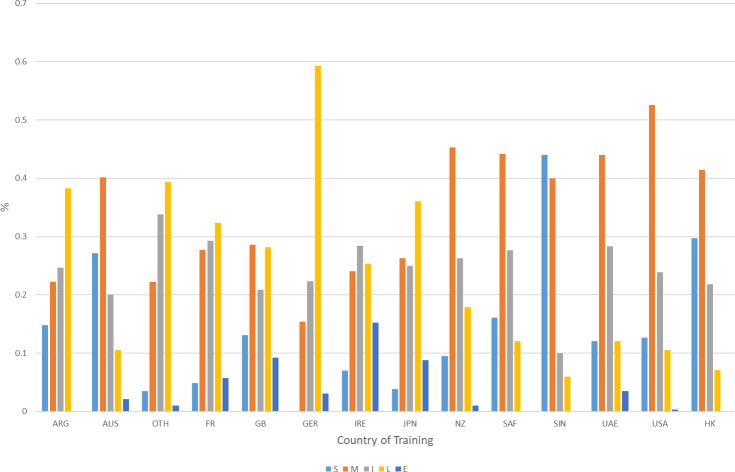
Percentage representation of horses successful in individual distance categories based on country of training. S: sprint 1000–1300 m (5–6.5 f, where f abbreviates furlongs) and sprint (CAN/USA) 1000–1599 m (5–7.99 f). M: mile 1301–1899 m (6.51–9.49 f) and mile (CAN/USA) 1600–1899 m (8–9.49 f). I: intermediate 1900–2100 m (9.5–10.5 f). L: long 2101–2700 m (10.51–13.5 f). E: extended 2701 m + (13.51 f + ).

Substantial differences in success at different distances can also be found with regard to the country of training (Fig. 4).

It is conspicuous that some countries are specialists in specific distances: there are considerable differences between the individual countries of training when it comes to the proportion of horses that are successful in races over particular distances. AUS, HK and SIN have the greatest numerical representation at shorter distances as countries of training. Horses trained in the USA and SAF most frequently achieve a high rating at the mile distance, and horses trained in GER, ARG and JPN are predominantly successful at the long distance. IRE, FR and GB are the most balanced in terms of the frequency of distance categories among successful horses.

**Figure 5:**
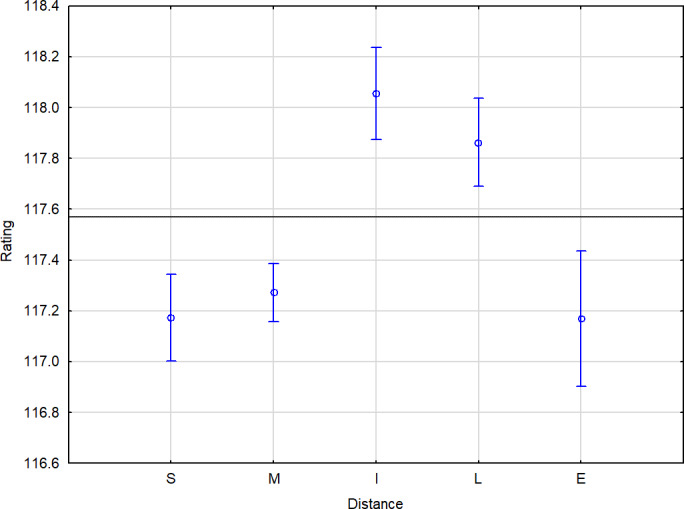
Performance summary of horses according to distance. The horizontal line denotes the mean rating of the monitored population. The vertical bars represent the standard errors of differences (confidence interval 0.95). S: sprint 1000–1300 m (5–6.5 f) and sprint (CAN/USA) 1000–1599 m (5–7.99 f). M: mile 1301–1899 m (6.51–9.49 f) and mile (CAN/USA) 1600–1899 m (8–9.49 f). I: intermediate 1900–2100 m (9.5–10.5 f). L: long 2101–2700 m (10.51–13.5 f). E: extended 2701 m + (13.51 f + ).

*Statistical analysis.* Statistically highly convincingly better ratings are achieved by horses at the intermediate (118.06) and long (117.86) distances compared to all other distances (Fig. 5).

### 3.6 The effect of the sire

*Evaluation of population.* When evaluating the results, it is important to take into account the fact that they are affected by the unequal length of time that individual stallions have spent in breeding programmes between 2004 and 2022. Some stallions have already finished their breeding careers, while others have only just begun. In total, of the 1056 sires registered in the database, 30 % have only 1 offspring and 14.3 % have 10 or more offspring. Three exceptional sires had more than 100 offspring in the IFHA databases during the monitored period, and 10 sires had more than 50 offspring (Table 5). Four of the 10 most frequently represented sires (1, 4, 9, 10) belong to the Sadler's Wells (USA) paternal line.

**Table 5:** List of the most successful sires in terms of number of offspring.

		Number of offspring	Avg rating of offspring
1.	Galileo (IRE)	193	119.00
2.	Deep Impact (JPN)	136	117.76
3.	Dubawi (IRE)	131	118.06
4.	Montjeu (IRE)	64	118.80
5.	Pivotal (GB)	61	117.64
6.	Dansili (GB)	58	117.69
7.	Shamardal (USA)	57	117.84
8.	Danehill (USA)	56	118.55
9.	Sadler's Wells (USA)	54	117.72
10.	High Chaparral (IRE)	50	117.96

**Figure 6:**
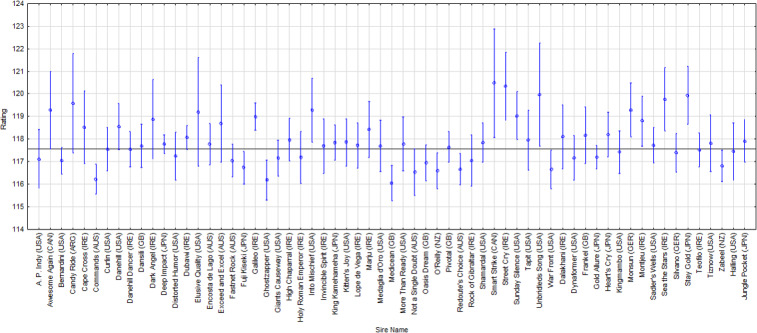
Performance summary of horses according to sire. The horizontal line denotes the mean rating of the monitored population. The vertical bars represent the standard errors of differences (confidence interval 0.95).

*Statistical analysis.* Although a statistically highly significant influence of the sire on the rating value was established by the multifactorial analysis, the Scheffé test did not show individual differences. Figure 6 shows the differences in the performance of the offspring of individual stallions and Table 6 lists all sires whose progeny achieved an average rating above 119.00. Five of these stallions (1, 2, 3, 6 and 10) belong to the Mr. Prospector paternal line (USA), which shows the exceptional quality of this sire line.

**Table 6:** List of the most successful sires in terms of mean performance of offspring.

		Avg rating of offspring	Number of offspring
1.	Smart Strike (CAN)	120.48	23
2.	Street Cry (IRE)	120.34	47
3.	Unbridled's Song (USA)	119.96	25
4.	Stay Gold (JPN)	119.94	32
5.	Sea the Stars (IRE)	119.76	45
6.	Candy Ride (ARG)	119.59	22
7.	Awesome Again (CAN)	119.29	28
8.	Monsun (GER)	119.28	39
9.	Into Mischief (USA)	119.27	22
10.	Elusive Quality (USA)	119.20	20
11.	Sunday Silence (USA)	119.03	35
12.	Galileo (IRE)	119.00	193

When the ratings of the offspring of individual sires are combined with their distance qualities, it is evident that some sires have produced offspring with a clearly defined successful distance, while others have offspring that are very balanced in performance over several distances (Table 7).

**Table 7:** Mean rating and proportion of offspring of sires at individual distances.

	S	M	I	L	E
Awesome Again (CAN)	116.00	118.86	120.33	116.00	No repr.
	3.57%	50.00%	42.86%	3.57%	0.00%
Candy Ride (ARG)	117.50	119.00	121.67	No repr.	No repr.
	9.09%	63.64%	27.27%	0.00%	0.00%
Danehill (USA)	116.50	117.20	118.32	120.41	117.67
	3.57%	26.79%	33.93%	30.36%	5.36%
Dansili (GB)	117.00	116.83	117.41	119.60	115.00
	3.45%	39.66%	29.31%	25.86%	1.72%
Deep Impact (JPN)	116.00	117.17	117.74	118.31	117.40
	0.74%	26.47%	27.94%	37.50%	7.35%
Dubawi (IRE)	117.58	117.84	118.97	118.03	116.20
	9.16%	38.17%	22.90%	25.95%	3.82%
Elusive Quality (USA)	118.33	118.44	126.00	115.00	No repr.
	30.00%	45.00%	15.00%	10.00%	0.00%
Galileo (IRE)	115.00	119.55	120.14	118.55	117.68
	0.52%	17.10%	26.42%	41.45%	14.51%
High Chaparral (IRE)	115.00	118.10	119.35	116.19	120.50
	4.00%	20.00%	40.00%	32.00%	4.00%
Into Mischief (USA)	117.86	119.73	120.50	No repr.	No repr.
	31.82%	50.00%	18.18%	0.00%	0.00%
Monsun (GER)	No repr.	122.00	120.57	118.71	117.75
	0.00%	10.26%	17.95%	61.54%	10.26%
Montjeu (IRE)	No repr.	116.50	118.75	119.71	117.08
	0.00%	6.25%	18.75%	54.69%	20.31%
Pivotal (GB)	116.40	117.96	118.29	117.30	115.00
	16.39%	37.70%	27.87%	16.39%	1.64%
Sadler's Wells (USA)	No repr.	121.00	117.56	117.45	118.13
	0.00%	1.85%	16.67%	53.70%	27.78%
Sea the Stars (IRE)	No repr.	119.75	120.00	120.11	118.90
	0.00%	17.78%	17.78%	42.22%	22.22%
Shamardal (USA)	119.14	117.22	117.95	118.25	No repr.
	12.28%	40.35%	33.33%	14.04%	0.00%
Smart Strike (CAN)	120.00	118.75	124.20	121.00	No repr.
	4.35%	52.17%	21.74%	21.74%	0.00%
Stay Gold (JPN)	No repr.	118.00	119.25	122.08	118.36
	0.00%	12.50%	12.50%	40.63%	34.38%
Street Cry (IRE)	117.29	120.50	122.93	117.33	No repr.
	14.89%	42.55%	29.79%	12.77%	0.00%
Sunday Silence (USA)	117.00	117.67	118.80	120.06	119.33
	5.71%	25.71%	14.29%	45.71%	8.57%
Unbridled's Song (USA)	115.50	118.88	126.00	117.00	No repr.
	8.00%	68.00%	20.00%	4.00%	0.00%

No repr.: no representation.

## 4 Discussion

### 4.1 The effect of the sex

The greatest number of stallions was found at the beginning of our monitoring in 2004 (73.04 %), but since 2007 their share has never exceeded 60.00 % and at the same time has not fallen below 54.00 %. The opposite development can be seen in geldings, which in 2004 comprised only 11.27 % and since 2005 have never fallen below 20.00 %. The greatest representation in the databases was achieved by geldings in 2018 (31.08 %). More (1999) also demonstrated the numerical superiority of males among racehorses in Australia (56.20 %) and even more so in the group of horses with higher earnings (66.60 %). Bolwell et al. (2014) claimed that 55.07 % of starters in flat races in New Zealand were male. According to the official statistics of individual countries (HRI, 2022; BHA, 2022) and research by other authors (Morrice-West et al., 2022; Velie et al., 2013), geldings predominated in the total number of horses being trained in all countries, followed by mares, while stallions formed the smallest share. The difference in the percentage of individual sexes within the entire population of horses being trained and according to the results for elite horses reflected the proven lower rating of mares and geldings.

The performance superiority of males in racing was also proven in studies by other authors (Mota et al., 1998; Ekiz et al., 2005; Entin, 2007; Sobczyńska, 2011; Velie et al., 2015; Özen et al., 2021). According to Kopečná et al. (2017), the reason for this could be the fact that there is significant pressure on the colts to become breeding stallions and they need to have the highest performance to be selected for a stud. According to our study, there was a decrease in the number of stallions in races after the age of 4, and their rating results also decreased. This pressure is strongest among 3- and 4-year-old horses, where we found an average rating of 118.06 and 118.15 respectively, with the performance subsequently declining. By contrast, geldings only achieved the highest number and rating at 5 and 6 years of age, and from this age on they also have a higher rating than stallions of the same age.

There are a number of potential reasons why, despite the established lower weight allowance, the mares achieved a lower rating than the stallions. Mukai et al. (2003) demonstrated that male horses exhibited lower heart rates at an identical running speed, suggesting that males possess a higher aerobic capacity. Seder and Vickery (2003) found that a filly typically had a shorter stride than a colt of the same age at the same velocity. Sobczyńska (2007) suggested that the relatively poor performance of mares may also reflect the distinct psychological traits (willingness to compete and win) of the sexes during racing. Pryor and Tibary (2005) mentioned that the different phases of the oestrous cycle could be one of the reasons for unwanted behaviour or poor performance by mares. According to Losecaat Vermeer et al. (2016), growing evidence from animal research suggests that testosterone modulates the motivation to compete due to its actions on the meso-corticolimbic dopaminergic system. Several other studies have looked at the association between testosterone levels and superior performance by horses and humans (Casto et al., 2020; Knight et al., 2022; Andersen et al., 2016), but the results are not uniform and more studies will be required to prove or disprove this theory in horse racing.

### 4.2 The effect of age

The number of horses in the database decreased in accordance with the age of the horses. Horses that are 3 to 5 years old make up 77.01 % of the top-performing population. Our results showed that the number of stallions and mares included in the IFHA database dropped very rapidly between 4 and 6 years of age. The mean age was 4.05 years for mares and 4.16 years for stallions. On the other hand, the proportion of geldings rose up until the age of 6, and their mean age was 5.34 years. It was not possible to identify the specific causes of this in the current study because we were not able to determine whether the horses ended their careers for breeding purposes or some of the stallions moved into the gelding group through castration or they were simply no longer ranked among the top performers at an older age. Özen et al. (2021) mentioned that career termination can occur for a variety of reasons, including veterinary advice on disease or injury as well as economic considerations and an appraisal of athletic ability. Sobczyńska (2007) suggested that females were more likely to be withdrawn from training and used for breeding purposes when training or health problems occur. The results obtained by Sharman et al. (2022) supported the idea of the most efficient horses being removed for breeding at a very early stage. They identified significant negative effects of advancing maternal and paternal age on offspring speed. According to their study, the ideal pairing to produce the fastest racehorses was a 6-year-old dam and a 4-year-old sire.

The transfer of the best stallions and mares into breeding programmes and performance decline were certainly important contributing factors; however, the health of the horses could also play a role. The age of the horse has been associated with the risk of injury in a number of studies (Williams et al., 2001; Lyle et al., 2011; Georgopoulos and Parkin, 2017).

According to More (1999), only 46 % of the horses that first started as 2- and 3-year-olds continued racing for 2 years after their first start in Australia. Similar results were reported by Sobczyńska (2007). In Poland 28 % of males and females ended their careers after the first racing season, whereas as many as 60 % of females and 48 % of males stopped racing after the second season. Other authors have also claimed that the mean career length of Thoroughbred horses is short: 14.70 months in Australia (Velie et al., 2013) and 17.79 months in Turkey (Özen et al., 2021).

At the same time, our results proved that mean performance also decreased with increasing age of racehorses. The effect of age on the performance of racehorses was also confirmed by Velie et al. (2014) with respect to several indicators (finish time, number of wins, place percentage, etc.). According to Takahashi (2015), the average speed of Thoroughbreds in Japan increased up until the age of 4.5 years. The effect of increased carry weight on average speed was small, and average speed increased as the horse grew. After the age of 4.5 years, the horses' average speed remained almost constant, with little variation. Gramm and Marksteiner (2010) found that a typical horse's peak racing age was 4.45 years. The rate of improvement from age 2 to age 4.5 was greater than the rate of decline after age 4.5. Ekiz et al. (2005) stated that the tendency to improve with age until 6 years of age and then decline was observed for racing time, best racing time and annual earnings in Turkey.

### 4.3 The effect of the country of foaling and country of training

According to the IFHA (2019), the percentage distribution of races between the continents was 40.03 % in Asia, 39.21 % in the Americas and 20.76 % in Europe. From the point of view of Thoroughbred breeding, the distribution between the continents was 35.90 % in Asia, 37.30 % in the Americas and 26.80 % in Europe.

In terms of prize money, the top prizes were distributed as follows: 56.48 % to Asia, 28.55 % to the USA and 14.97 % to Europe. From a long-term perspective, the number of races in individual regions was unchanged, but the prize money situation was increasingly in favour of Asia. Individual regions and countries have their own specific history of Thoroughbred breeding and the development of horse racing, and this is reflected in the different success rates of their horses on a global scale.

Our results showed differences between individual countries in terms of the quantitative and qualitative evaluation of international horse ratings. The influence of the country of origin on the performance parameters of racehorses in Hong Kong was demonstrated by Velie et al. (2015). They mentioned the following as possible reasons: genetic differences between regions of origin, environmental differences between regions, or even differences in the way horses were selected for transfer to Hong Kong. The breeding emphasis in certain racing regions (e.g. sprinters vs. stayers) means that horses from those regions are better equipped to perform well. In a similar vein, Williamson and Beilharz (1998) stated that in Australia, unlike in Europe, a horse is tested during the year on a range of distances from 1200 to 2400 m, while in Europe the focus tends to be on one distance category. As a result, the nonspecialist horses cannot compete with specialists in a particular distance in the international field.

Our study focused on comparing the results of individual countries, but for a more detailed specification of the reasons for these differences, further research encompassing more influencing factors is necessary.

**Table 8:** Comparison of the percentage representation of horses and the mean rating of horses from individual countries according to our results and the IFHA Statistics TJCIS (2022) and IFHA (2019).

	2021	2019	2022	2022	Rating –	Rating –
	group	foals ${}^{2}$	country	country	country	country
	races ${}^{1}$		of foaling	of training	of foaling	of training
ARG	161	6.84%	3.34%	2.74%	116.22	115.88
AUS	334	14.15%	13.98%	16.41%	117.14	117.01
FR	116	6.17%	4.86%	6.08%	117.71	118.12
GB	160	5.19%	10.94%	19.76%	117.93	118.05
GER	43	0.79%	1.52%	1.22%	117.50	117.16
IRE	73	10.16%	20.36%	4.86%	117.99	180.80
JPN	129	8.05%	14.89%	15.81%	117.68	117.65
NZ	91	3.81%	3.65%	0.00%	116.90	116.35
SAF	114	2.55%	3.34%	3.65%	116.93	116.80
USA	437	21.78%	21.28%	21.88%	117.73	117.58

${}^{1}$ TJCIS (2022). ${}^{2}$ IFHA (2019).

The largest number of group races was run in the USA and, according to the IFHA (2019), more than a fifth of Thoroughbred horses were born there. According to our results based on the databases of the most successful horses, the USA had numerical superiority in terms of both horses born and trained there.

From the point of view of quality, horses born and trained in Ireland were statistically proven to have achieved the best ratings. The exceptionality of Ireland is also underlined by the data from the IFHA (2019) and TJCIS (2022) statistics because in terms of the number of foals born, the number of horses trained and the number of group races, Ireland does not even belong to the countries with the highest share (Table 8). Nevertheless, based on the IFHA databases, 20 % of the best-rated horses in the world were born in Ireland. According to Kopečná et al. (2017), this is due to the long history of breeding and training Thoroughbred horses, their excellence in managing the stud and training yards, and, last but not least, the quality of the trainers. The exceptional position of horse racing in Ireland is borne out by the HRI statistics (HRI, 2018, 2023): compared to other countries, Ireland has approximately 50 Thoroughbred horses per 10 000 people, and over the last 5 years, foreign-trained horses won less than 5 % of the prize money in Ireland.

When the proportion of foals born in 2019 was compared with the proportion of top-performing horses in 2022, in addition to Ireland's success, there was also a high success rate for horses from Great Britain and Japan.

According to our results, Great Britain was the second most prolific country in training successful racehorses, and horses born there achieved the second-highest average rating for the period under review. There was also a strong link with Ireland, as Irish-bred horses made up 41.70 % of horses trained in Great Britain.

According to the HS (2019), British-trained horses won 37 Group 1 flat races outside of Britain in 2018. To put this in context, the country with the second-highest number of overseas winners was Ireland, with 16.

Some courses around the world are so keen to attract top-quality British horses to race in their meetings that they offer enormous incentives. In 2018 the Japan Racing Association offered to provide a USD 1 000 000 bonus, in addition to help with transportation costs and support in Japan, to any horse that wins the prestigious Group 1 King George VI and Queen Elizabeth Stakes at Ascot or York's Juddmonte International and then goes on to win the Japan Cup (HS, 2019). Financial motivation is one of the methods that has contributed to the promotion and improvement in the quality of Thoroughbred breeding and horse racing in Japan. An improvement in the racing performance of horses in Japan was recognized by Oki and Sakaki (1996). Their results also suggested that the Thoroughbred horses in Japan have improved their racing time and that imported sires played an important role in this improvement. In Japan, great emphasis is also placed on research into the performance of racehorses in terms of genetic predispositions and heritability (Moritsu et al., 1994; Oki et al., 1994; Tozaki et al., 2012; Kakoi et al., 2018; Fawcett et al., 2019).

It is evident from Table 9 that each country retained the majority of its native born horses, but the proportion of horses imported from abroad varied widely. These results showed the popularity of Irish horses in Great Britain, France and as far away as the USA. Japan, on the other hand, had a more closed racing system, and the largest foreign share in the country's horses was held by the USA.

**Table 9:** Percentage share of the most successful horses in individual countries of training based on their country of foaling.

	ARG	AUS	FR	GB	GER	IRE	JPN	NZ	SAF	USA
ARG	100.00%	0.00%	0.00%	0.00%	0.00%	0.00%	0.00%	0.00%	0.00%	0.00%
AUS	0.00%	71.59%	1.34%	4.47%	0.89%	4.36%	1.23%	15.21%	0.00%	0.89%
FR	0.00%	0.22%	39.69%	21.51%	2.22%	28.38%	0.22%	0.00%	0.00%	6.87%
GB	0.00%	0.41%	4.82%	43.75%	0.61%	41.70%	0.00%	0.10%	0.10%	8.50%
GER	0.00%	0.00%	0.77%	10.77%	77.69%	10.00%	0.00%	0.00%	0.00%	0.77%
IRE	0.00%	0.78%	1.29%	16.02%	0.52%	71.58%	0.52%	0.52%	0.00%	8.79%
JPN	0.00%	0.14%	0.00%	0.29%	0.43%	0.43%	96.01%	0.14%	0.00%	2.57%
NZ	0.00%	8.42%	0.00%	0.00%	0.00%	1.05%	0.00%	90.53%	0.00%	0.00%
SAF	1.01%	9.55%	0.00%	0.50%	0.00%	1.01%	0.00%	0.00%	86.43%	0.00%
USA	0.91%	0.00%	0.97%	2.33%	0.39%	2.85%	0.32%	0.13%	0.00%	88.23%

Lines: country of training; columns: country of foaling.

The average rating of horses from individual countries was affected by the previously confirmed difference in the performance of horses of different sexes, and there were noticeable differences between individual countries of training when it came to the percentage of successful horses of each sex (Table 10). In most countries stallions were the most frequently represented, but in Australia, South Africa and New Zealand geldings predominated numerically.

**Table 10:** Percentage share of the most successful horses in individual countries of training based on sex.

	Stallion	Mare	Gelding
ARG	96.30%	3.70%	0.00%
AUS	34.23%	12.75%	53.02%
FR	62.53%	24.39%	13.08%
GB	61.17%	14.65%	24.18%
GER	85.38%	7.69%	6.92%
HK	7.63%	0.00%	92.37%
IRE	72.61%	19.64%	7.75%
JPN	89.44%	9.13%	1.43%
NZ	28.42%	17.89%	53.68%
OTH	88.89%	6.57%	4.55%
SAF	44.22%	6.53%	49.25%
SIN	14.00%	4.00%	82.00%
UAE	65.25%	3.55%	31.21%
USA	60.74%	24.26%	15.01%

### 4.4 The effect of the race distance

The results of research by Williamson and Beilharz (1998) in Australia showed that heritability of speed, stamina and best distance were high. A genetic study by Hill et al. (2010) identified an *MSTN* (myostatin gene) sequence polymorphism that was strongly associated with best race distance among elite racehorses. Rooney et al. (2018) provided mechanistic evidence that a SINE (short interspersed nuclear element)insertion uniquely accounted for the *MSTN* “speed gene” effect on race distance aptitude in the Thoroughbred horse. This offers breeders and trainers the opportunity to better estimate the ideal distance for their horses to race and thus improve their horses' chances and boost their performance and rating. In addition to genetic predispositions, a horse's results are influenced by external factors and competition.

According to a definition from the IFHA (2022), the ratings are based on the performance of horses running worldwide in elite races held during the designated period, taking into account the quality of the opposition and the achievements of each horse. Our study confirmed the preference for races at the classic race distances, because at these distances the horses achieved the highest number and were statistically proven to have the highest rating. Therefore, these distances should represent the most competition and the core of Thoroughbred breeding throughout the world. This assumption is also confirmed by Kopečná et al. (2017), who find a statistically significantly higher rating at intermediate and long distances in the population of 3-year-old horses.

Our study demonstrated the numerical and performance superiority of stallions at all distances except the sprint. According to our findings, geldings had a higher mean rating in sprint races than mares and stallions, and at the same time, geldings aged 5 years or older were the most numerous group at the sprint distance (40.42 %). Better performance by older horses over short distances was demonstrated by Moritsu et al. (1994) and Mota et al. (1998). These findings could be influenced by the fact that the best-performing colts and fillies are prioritized over classic race distances, and older horses therefore have less competition in the sprint.

As mentioned in the results, the most common distance for mares was the mile, and as the distance increased, their number decreased. According to Takahashi (2015), the ratio of males to females was equal across shorter distances in Japan but increased along with race distance; the ratio was almost 2:1 for 2000 m races on turf and 1800 m races on dirt. The reason for the lower number of mares starting at longer distances could be the previously mentioned issue of the competitiveness of mares, which decreases as the race distance increases. As is supported by the results of other studies, Moritsu et al. (1994) demonstrated better racing times for males at the distance of 1800 m but not at the distance of 1200 m in Japan. In addition, Entin (2007) found that intact males were statistically significantly 0.7 % faster than females at distances shorter than 1609 m and 1.4 % faster at distances longer than 1609 m.

### 4.5 The effect of the sire

Research into horse populations has been used to examine the heritability of racing performance, and it is now recognized that many measures of racing performance (particularly race speed and ratings) are heritable (More, 1999). Numerous studies have focused on the genetic influence on racing performance and the calculation of heritability coefficients for individual performance indicators (Moritsu et al., 1994; Mota et al., 1998; Williamson and Beilharz, 1998; Sobczyńska and Lukaszewicz, 2004; Taveira et al., 2004; Svobodova et al., 2005; Bakhtiari and Kashan, 2009; Thiruvenkadan et al., 2009; Bugislaus, 2010; Tozaki et al., 2012; Bower et al., 2013; Velie et al., 2014; Bailey et al., 2022).

Other authors have examined inbreeding and genetic diversity among Thoroughbreds (Cunningham et al., 2001; Binns et al., 2011; Todd et al., 2018; McGivney et al., 2020; Bailey et al., 2022; Hill et al., 2022). A study by Cunningham et al. (2001) confirmed the narrow genetic base of the Thoroughbred and provides a comprehensive analysis of the contributions made by foundation animals. Seventy-eight percent of alleles in the current population are derived from 30 founders, 27 of these being male. Ten foundation dams account for 72 % of maternal lineages, while one foundation sire (Darley Arabian) is responsible for 95 % of paternal lineages.

According to the results obtained by Todd et al. (2018), over 80 % of inbreeding in the contemporary population is accounted for by a small number of ancestors from the foundation of the breed. As a result of many generations of inbreeding, the average F of the 21st century Thoroughbred population is 0.139 ( s=0.011 ).

Todd et al. (2018) emphasized that inbreeding to these ancestors has variable effects on fitness, demonstrating that an understanding of the distribution of genetic load is important in improving the phenotypic value of a population in the future. Extensive genetic research by McGivney et al. (2020) has shown that the global Thoroughbred population has a small effective population size and that a limited number of stallions have had a disproportionate influence on the genetic composition of the Thoroughbred; 97 % of the pedigrees of the horses included in their study featured the ancestral sire Northern Dancer (CAN), and 35 % and 55 % of the pedigrees in Europe and Australasia contained Sadler's Wells (USA) and Danehill (USA) respectively. Our results confirmed the increased number of descendants of these sires in the population of the most successful Thoroughbred horses in the last 20 years, even though both of them are already dead. Both sires have successors in breeding programmes, and Sadler's Wells' son Galileo (IRE) is considered an even more important sire than his father. In the IFHA 2004–2022 databases, his offspring formed the largest group, and their performance also proved his current status as the most successful sire (see Tables 4 and 5). Our results also confirmed the exceptionality of the Mr. Prospector (USA) sire line. The successors of this sire line occupied the top three positions with regard to the highest mean rating of offspring.

Our results confirm the previous research and the importance of sire selection, but we consider this part of the results to be only preliminary research and monitoring of the current situation, as not all of the offspring of individual stallions have been taken into account and the influence of the mare has not been examined either. Only a further, more extensive study taking into account the aforementioned factors would produce convincing results that would make it possible to draw conclusions about the breeding results of sires.

In the next decade, it will be possible to assess whether the next generation of sires will achieve the qualities of their ancestors and whether breeders will make use of the results of genetic studies and respond to scientists' warnings about inbreeding. However, the fact that most of the current sires can be traced to the same sire lines confirms the statement by McGivney et al. (2020) that there has been a highly significant increase in inbreeding in the global Thoroughbred population during the last 5 decades, which is unlikely to be halted due to current breeding practices.

According to McGivney et al. (2020), genomics-based measures using high-density genome-wide SNP (single-nucleotide polymorphism) information and a large reference population are likely to offer the best opportunity to slow and reverse the potential effects of inbreeding.

## 5 Conclusions

Thoroughbred racing and breeding have a long historical tradition and are a permanent part of most countries and cultures. This is a worldwide phenomenon in terms of the expansion of horse racing, the unified evaluation system and Thoroughbred breeding methods. The aim of this study was to monitor the current situation in international horse racing during the last 19 years and to confirm statistically significant differences in terms of age, sex, the sire of the horse, race distance, country of foaling and country of training.

Statistical analysis established a significant ( p<0.001 ) influence of all observed factors. In terms of sex, the highest ratings and numerical superiority were achieved by stallions. On the other hand, there was no significant difference between the ratings of mares and geldings.

Stallions and mare that were 3 to 4 years old made up more than half of the population, and in keeping with expectations, achieved the best performance, after which the performance of the horses still participating in racing declined.

Despite the downward trend, horses born in the USA still have a numerical advantage; second place is held by Ireland, and the number of racehorses from Japan exhibited an upward trend. In terms of performance, there are highly conspicuous differences between countries, and the best horses are from Ireland and Great Britain. During the time period of our analysis, a decrease in the number of successful horses trained in the USA and in France was also evident. In contrast, the number of horses trained in Australia and Japan increased. The best rating was achieved by horses trained in Ireland, which are statistically significantly more successful than horses trained in all other countries except Great Britain and France.

The racing horses achieved the highest number in the mile distance category and were statistically proven to have the highest rating for the intermediate and long distances.

The findings of this study contribute to a better understanding of the Thoroughbred horse's racing performance and will help breeders and riders to evaluate current trends in international racing and put these data to practical use. However, for an objective assessment of performance and progress in the breeding of Thoroughbreds, more studies of the total population of horses involved in racing are necessary.

## Data Availability

The data that support the findings of this study are available from the corresponding author upon reasonable request.
